# Vitamin E: Where Are We Now in Vascular Diseases?

**DOI:** 10.3390/life12020310

**Published:** 2022-02-18

**Authors:** Anahita Garg, Jetty Chung-Yung Lee

**Affiliations:** School of Biological Sciences, The University of Hong Kong, Pokfulam Road, Hong Kong, China; anahita@connect.hku.hk

**Keywords:** vitamin E, tocopherol, antioxidant, oxidation, cardiovascular diseases, atherosclerosis, low-density lipoprotein

## Abstract

Vitamin E is one of the most popular fat-soluble vitamins in pathological research and has been under scrutiny since the 1980s as a vital dietary component of food. The antioxidant effect of vitamin E has been widely studied due to its benefits in the prevention of various cardiovascular diseases. In recent years, alternative effects of vitamin E, in terms of anti-inflammatory pathways and gene regulation, have also been of interest to researchers. This review examines the role of dietary vitamin E (α-tocopherol) as an antioxidant and bioactive molecule in promoting vascular health. While the antioxidant effect of vitamin E is well established, knowledge about its capacity as a promising regulatory molecule in the control of the vascular system is limited. The aim of this review is to discuss some of these mechanisms and summarize their role in the prevention of cardiovascular diseases (CVD). Here, we also briefly discuss foods rich in vitamin E, and deliberate some potential toxicological effects of excessive supplemental vitamin E in the body.

## 1. Introduction

Vitamin E has historically been viewed as an important antioxidant in the body. However, its functions as a regulatory molecule were only proposed in the late 1980s and early 1990s [1]. Vitamin E is classified as a vitamin since it is an organic nutrient required by the body in small amounts to maintain its normal physiological function. A deficiency of vitamin E can lead to ataxia and also increase the risk of developing other diseases due to fat malabsorption [2].

**Figure 1:**
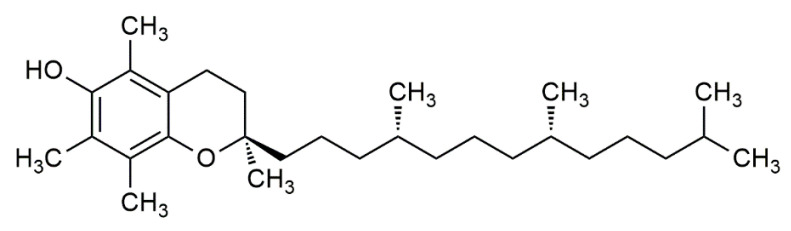
The chemical structure of α-tocopherol (αT).

**Figure 2:**
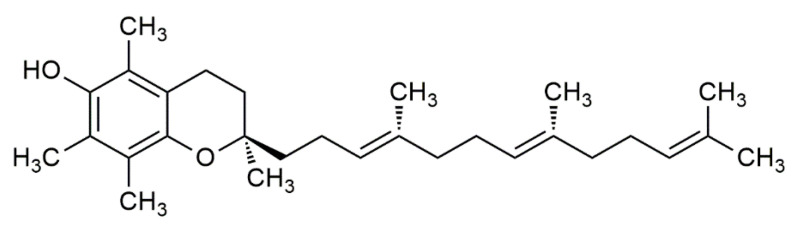
The chemical structure of α-tocotrienol.

In 1922, vitamin E was identified by Evans and Bishop as an organic lipid-soluble compound capable of restoring reproduction in diet-restricted sterile rodents. These rodents were fed foods rich in vitamin E, which was then noted as ‘substance X’, and due to its biological effects, it earned the name “tocopherol”, meaning “to bring offspring” in Greek [3]. Thereafter, the term vitamin E was adapted to represent tocochromanols in plant lipids, which are composed of a hydroxylated chromanol ring attached to a hydrophobic phytyl side chain. Tocochromanols include both tocopherols and tocotrienols (Figure 1 and Figure 2), which differ in the saturation of their phytyl side chains. The former has a saturated phytyl (isoprenoid) side chain, while the latter has an unsaturated phytyl side chain with 3 double bonds. Both tocopherol and tocotrienol have four vitamers each, and are noted as α, β, γ or δ, such that they can be distinguished based on the extent and position of methyl groups on the chromanol ring. Despite the existence of multiple tocopherol and tocotrienol vitamers, where some have been found to show biological activities, the attribute of ‘vitamin’ is only given to α-tocopherol (αT) due to its selective uptake [4] and poor recognition of other vitamers in the liver by the α-tocopherol transfer protein (α-TTP) [5]. This was further supported by the fact that only α-tocopherol (with RRR stereochemistry) could prevent diseases linked with vitamin E deficiency. Hence, other vitamers of tocopherol and tocotrienol are not considered as ‘vitamin E’ in nutritional studies [2], but rather are noted as its analogues or derivatives.

Unlike the case of other vitamins, the vitamer αT cannot be interconverted in the body, i.e., it cannot use other vitamers of tocopherols or tocotrienols to make αT [6]. Moreover, αT cannot be synthesized in the human body, making it an essential nutrient. This makes it crucial to consume foods that are specifically rich in αT to ensure that one does not suffer from vitamin E deficiency [7]. While vitamin E deficiency in humans is rare, it can occur due to hampered intestinal nutrient absorption; in particular, via fat malabsorption through severe malnutrition, cystic fibrosis, chronic diarrhea, chronic pancreatitis, liver disease, etc. [6]. Severe vitamin E deficiency can also lead to neurological disorders and damage the central nervous system, such as axonal dystrophy, muscular lesions, and other neuromuscular complications [4,8,9]. 

## 2. Foods Rich in α-Tocopherol

The recommended dietary allowance (RDA) for vitamin E is 15 mg/day for men and women, and 19 mg/day for lactating women [10]. The upper level of intake (UL) is 1000 mg/day, since an intake equal to or greater than this level can cause haemorrhages [5]. However, it is virtually impossible to achieve this level through the natural intake of tocopherols. A study on the estimated average requirement (EAR) of important micronutrients showed that while most Americans manage to meet their need for many nutrients, significant parts of the population, especially children, have intakes below the required EAR for dietary vitamin E. When supplementation of vitamin E was also considered in the study, the intake below the EAR reduced from 91% to 60%, indicating that the American population relies more on supplementation and fortification rather than on foods that are naturally enriched with tocopherols to meet their daily vitamin E requirement [11]. 

Vitamin E is found in a variety of foods, and is abundant in nuts, seeds, and their oils (Table 1). The highest amount of αT is found in wheat germ oil, followed by almonds, sunflower oil, safflower oil, hazelnuts, and peanuts [10,12]. In our daily diet, vitamin E is generally obtained from eggs, meat, fish, margarine, bread, green leafy vegetables, fruits, and fortified cereals [13]. Vitamin E is exclusively found in the αT form in sunflower seeds, contributing to its use as a valuable dietary source. Furthermore, a few trials showed that nut consumption rich in vitamin E, especially hazelnuts and peanuts, was a major reason for cardioprotective effects [14].

Shahidi et al. contend that speciality and fruit seed oils are also rich sources of αT, and are underutilised as natural sources of vitamin E. These include pecan, raspberry, blackberry, grape, date, wild pear, sophia, and chia seed oils. Other important but lesser-known sources of αT include pseudo-cereals such as quinoa, amaranth and canihua, lentils, chickpeas, and regional fruits such as Murumuru, bacuri, cactus, jaboticaba, and navelwort [15]. The high percentage of both αT and unsaturated fatty acids in plants additionally suggests that it is the most important antioxidant molecule present in them, and can potentially prevent the oxidation of unsaturated fatty acids in plants. A range of different tocotrienols and tocopherols are also found in algae such as Chlorella, *Stichococcus bacillaris*, *Dunaliella salina*, and *Cladophora stichotoma*, among others [16]. This suggests that the use of algae as natural sources of vitamin E should be further explored in research. 

In addition, it should be noted that vitamin E is heavily interdependent on the presence of other vitamins and factors, namely vitamin B3, vitamin C, beta-carotene, thiols, glutathione, and selenium. This also suggests that a balanced diet needs the presence of other micronutrients to ensure an optimum amount of vitamin E is absorbed and metabolized by the body [10]. Certain foods, while not rich in vitamin E themselves, are used to protect αT present in lipoproteins. For example, caffeic acid, a phenolic compound found in coffee, protects αT from oxidation, as well as low-density lipoproteins (LDLs). Similarly, the long-term consumption of olive oil, which is also rich in phenolic compounds, improves the blood lipid profile, particularly high-density lipoproteins (HDLs), and enhances vitamin E’s capacity as an antioxidant in patients suffering from hypercholesterolemia [17].

**Table 1:** Foods rich in α-tocopherol (αT) found in our diet [12,18].

Food Category	Food	Milligrams (mg) per Serving	Percent Daily Value
	Wheat germ oil, 1 tablespoon	20.3	100
	Sunflower oil, 1 tablespoon	5.6	28
Oils	Safflower oil, 1 tablespoon	4.6	25
	Corn oil, 1 tablespoon	1.9	10
	Soybean oil, 1 tablespoon	1.1	6
	Sunflower seeds, dry roasted, 1 ounce	7.4	37
Seeds and Nuts	Almonds, dry roasted, 1 ounce	6.8	34
	Hazelnuts, dry roasted, 1 ounce	4.3	22
	Peanuts, dry roasted, 1 ounce	2.2	11
Processed food	Peanut butter, 2 tablespoons	2.9	15
	Spinach, boiled, ½ cup	1.9	10
	Broccoli, chopped, boiled, ½ cup	1.2	6
Fruits and Vegetables	Kiwifruit, 1 medium	1.1	6
	Mango, sliced, ½ cup	0.7	4
	Tomato, raw, 1 medium	0.7	4
	Spinach, raw, 1 cup	0.6	3

## 3. The Role of Vitamin E in the Pathogenesis of Cardiovascular Diseases

This review briefly summarises the role of vitamin E as an antioxidant and explores its function in regulating molecular activities. It focuses on pathological diseases of the vascular system, namely cardiovascular diseases (CVDs). There is a high incidence of CVD globally, which is also associated with the risk of morbidity. Studies based on Western populations described a high incidence of coronary heart disease owing to obesity, hypercholesterolemia, and atherosclerosis. Moreover, it is reported that Asian populations also suffer from long-term fatal CVDs due to risk factors such as hypertension, smoking, and increasing obesity [19]. While a positive effect of vitamin E on vascular health has been established [20,21], it is aimed to first briefly discuss its antioxidant effect, and then elaborate on its non-oxidative molecular functions in the vascular system. 

### 3.1. Antioxidant Mechanism

Clinical trials reported during the 1990s and early 2000s are reviewed here to gain better insights into the aetiology of vitamin E on the human vascular system that were established after its discovery.

**Figure 3:**
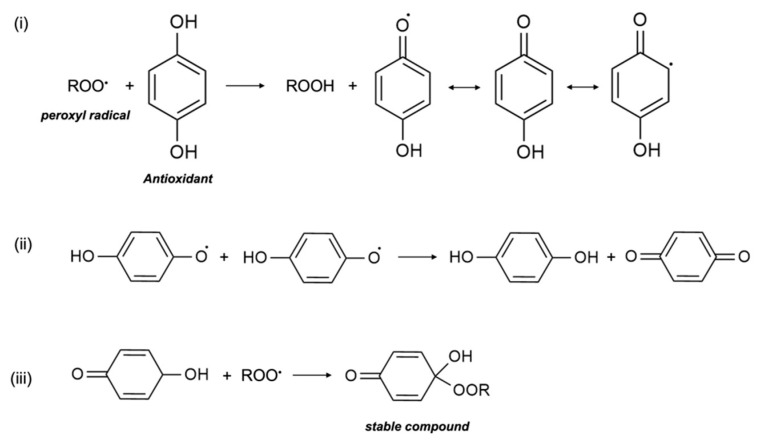
Termination of free radicals by antioxidants. The peroxyl radical is scavenged by antioxidants e.g., α-tocopherol. The resulting metabolite either (**i**) stabilizes itself, (**ii**) combines with another similar metabolite, or (**iii**) scavenges another peroxyl radical.

In in vitro research, vitamin E was shown to act as a scavenger of the peroxyl radical (LOO•), which helps to inhibit free-radical propagation and thereby lipid oxidation [1]. In cells, vitamin E is found in the phospholipid bilayer of the cell membrane and in plasma lipoproteins, and protects polyunsaturated fatty acids (PUFA) in situ from oxidation by peroxyl radicals. During the process of lipid peroxidation, molecular oxygen reacts with lipid carbon-centred radicals (L•) of various organic molecules such as fatty acids, cholesterol, phospholipids, etc., to form LOO•, which then reacts with PUFA to form lipid hydroperoxide (LOOH) and another L•. Antioxidants, including vitamin E, scavenge free radicals and break the lipid peroxidation chain reaction. In the presence of LOO•, the phenolic hydroxyl group of αT undergoes an event of propagation reaction and forms into a tocopheroxyl radical, leaving LOOH and preventing the development of L• (Figure 3). The tocopheroxyl radical is much more stable and harmless as compared to the peroxyl radical, and potentially converts into tocopherol via reduction from other antioxidants, such as ascorbic acid (vitamin C) or with another tocopheroxyl radical [22]. Vitamin E is an extremely efficient antioxidant since its rate of reaction and scavenging of LOO• is much higher than the rate of peroxidation of lipid molecules by peroxyl radicals [23]. In some cases, vitamin E can also act as a pro-oxidant and oxidize other lipids. Nonetheless, to date, the extent of vitamin E recycling and the conditions required for its antioxidant effect are not clearly known [5].

LOO• + αT → LOOH + αT • (tocopheroxyl radical)

The oxidative-modification hypothesis of atherosclerosis postulates that the accumulation of LDL in the sub-endothelium of arteries and its subsequent oxidation is the main cause of atherosclerosis. LDL accumulation initiates the recruitment of monocytes and macrophages, which further favors the peroxidation of LDL. The oxidized LDL generated is internalized by macrophages, which converts them into foam cells. Foam cells can accumulate in the sub-endothelium of arteries, which results in inflammation and plaque formation. The uptake of oxidized LDL by the scavenger–receptor pathway is not negatively regulated and leads to increased regulation of cholesterol. Chronic inflammation of endothelial cells and the narrowed lumen of arteries lead to the development of atherogenesis. Atherosclerotic arteries are a major developmental factor for various CVDs, since it narrows the lumen of arteries, cause hardening of the sub-endothelium, reducing blood flow and damaging endothelial cells. Furthermore, foam cells are cytotoxic in vascular cells, which leads to lipid and lysosomal enzyme release and vascular lesion formation. LDL oxidation and its cytotoxicity may be prevented by the incorporation of vitamin E, thus preventing the development of atherosclerosis, and subsequently reducing the risk of CVDs [24,25]. This is further discussed below and summarized in Table 2. 

Various clinical trials conducted in the late 1980s and 1990s perceived vitamin E to be a strong antioxidant that aided in preventing various CVDs. Followed by the hypothesis of αT’s antioxidant role in preventing LDL oxidation, several studies conducted large-scale human trials to investigate the reduction of various CVD risks. In 1993, separate studies (the Health Professional’s Follow-Up Study and the Nurses’ Health Study) were conducted in males and females to understand the relationship between vitamin E consumption and the risk of coronary artery disease (CAD). These trials confirmed that high oral doses of both dietary and supplemental (100–250 IU/day) vitamin E reduced the risk of major coronary diseases such as CAD, non-fatal myocardial infarction (MI), and cardiovascular death by 35–40%. These studies correlated the results with the reduction of cell-meditated oxidation of LDL by vitamin E and found reduced rates of atherosclerosis and restenosis [26,27]. In 1995, the Cholesterol Lowering Atherosclerosis Study conducted a subgroup analysis on the supplementary intake of vitamin E and the rate of progression of CAD. Researchers conducted angiography on the subjects after 2 years of intervention and found that supplementation of >100 IU/day of vitamin E caused the least progression in coronary artery lesions due to a lower rate of stenosis [28]. The Cambridge Heart Antioxidant Study (CHAOS) conducted in 1996 had similar angiographic findings. αT treatments were given to 2002 patients suffering from atherosclerosis for a median time of 510 days and showed a lower rate of non-fatal MI due to the reduced rate of macrophage-rich atherosclerotic lesion formation [29]. In vitro studies also showed a direct correlation between αT levels and oxidized LDL in the prevention of arterial dysfunction; high tissue levels of αT lowered the risk of endothelial dysfunction from oxidized LDL [30]. Another trial, known as the Secondary Prevention with Antioxidants of CVD in Endstage Renal Disease (SPACE) Study (2000), indicated that chronic hemodialysis patients have high vascular disease related mortality rates. Patients that undergo hemodialysis to treat renal diseases have elevated oxidative stress; however, vitamin E supplementation potentially prevented vascular complications, especially CVD. This observation suggests that vitamin E lower the rate of oxidized LDL production, which subsequently reduced the rates of non-fatal MI by 70%, cardiovascular mortality by 46%, and ischemic stroke by 14% [31]. A relatively recent clinical trial on the effect of vitamins on cardiovascular health in a Russian population showed that multivitamin supplementation, including αT, had an inverse relationship with the risk of CVDs. The Russian population was characterized by a high alcohol intake, smoking, and a low intake of natural vitamin sources such as fruits and vegetables, leading to poor antioxidant status. After continuous supplementation for 8 weeks, subjects showed vastly improved antioxidant levels, including αT levels, and a reduction in heart health risk biomarkers [32]. A study conducted in 2018 also showed that vitamin E supplementation significantly improved the vascular health of patients with Hp2-2 genotypes. Hp2-2 genes code for haptoglobin and increase the risk of endothelial dysfunction, MI, and type II diabetes, and is suggested to be regulated via vitamin E at a therapeutic level [33].

On the other hand, it has also been reported that the protective effects of tocopherol against LDL oxidation and its vascular function may not be due its antioxidant property. It was found in a long-term clinical trial that vitamin E may not have protective effects against CVD. One important study was the Alpha-Tocopherol, Beta-Carotene Cancer Prevention Trial (ATBC) conducted in 1996 on Finnish smokers. The effect of vitamin E on the prevention of angina pectoris was deemed unlikely to be of public health significance. Similar findings were shown in patients with intermittent claudication and those undergoing coronary angioplasty [34]. The Heart Outcomes Prevention Evaluation—The Ongoing Outcomes (HOPE-TOO) Study additionally reported that there was no significant difference in the incidence of major cardiovascular events after daily long-term administration of a high dose (400 IU) of natural vitamin E. In fact, it further suggested an increased risk of heart failure [35]. Similarly, the Women’s Health Study (WHS) conducted from 1992–2004 to test if vitamin E supplementation reduced the risk of CVD in women further showed a non-significant risk reduction after long-term administration of vitamin E supplementation [36]. The Physicians’ Health Study II conducted in 2008 among middle-aged and older men did not find any correlation between supplemental use of vitamin E and the risk reduction of non-fatal MI, angina, or heart failure [37]. This has also been supported by older studies conducted on a small-scale or population-subset level. Moreover, the MRC/BHF Heart Protection Study conceded that supplemental doses of vitamin E, along with other antioxidant vitamins, did not decrease the risk of incidence or mortality of CVD and cancer [38]. A recent study conducted on a Singaporean population contends that vitamin E supplementation did not have any preferential benefit in patients with the Hp2-2 genotype, but rather showed an increased risk of arterial stiffness in patients with high haptoglobin concentrations [39].

Due to the opposing views on the antioxidant effect of vitamin E in preventing various CVDs, it is recommended that more advanced clinical trials with larger test groups of participants should be investigated, with higher control on external factors such as age, eating patterns, environmental influences, etc. Additionally, a more quantitative assessment is needed to measure the oxidative stress of the human body to correctly prove the oxidation hypothesis. Moreover, the LDL oxidation rate in humans can be very slow, and atherosclerotic lesions can take many years to form. It is plausible that only a sub-population may respond to vitamin E supplementation, while some may be influenced by other environmental and genetic factors; for example, a family history of hypercholesterolemia [25]. Alternatively, an examination of the molecular roles of vitamin E in the prevention of cardiovascular complications could lead to new insights in its functions. 

**Table 2:** Summary of the antioxidant effects of vitamin E in vascular diseases.

Pathological Condition	Effect	Target Tissue/Organ	Reference
Atherosclerosis	↓ LDL oxidation, foam cell formation	Arteries	[24]
Non-fatal MI, CV death	↓ stenosis, ↓ atherosclerosis	Arteries, heart	[26,27]
Coronary artery disease	↓ stenosis, ↓ coronary artery lesions	Arteries	[28]
Non-fatal MI	↓ atherosclerotic lesion formation	Arteries	[29]
Arterial dysfunction	↓ LDL oxidation, × PKC	Arteries, endothelium	[30]
Secondary non-fatal MI, CV death (from chronic hemodialysis)	↓ LDL oxidation, ↓ atherosclerotic plaque, ↓ platelet aggregation, ↓ ischemic stroke	Vascular system	[31]
Endothelial dysfunction, MI, CVD	× antagonistic effect on Hp2-2 genotypes	Vascular system	[33]

↓: reduces rate of; ×: suppresses; LDL: low-density lipoprotein; MI: myocardial infarction; CV: cardiovascular; PKC: protein kinase C.

### 3.2. Potential Molecular Mechanisms

While vitamin E’s antioxidant mechanism is extremely well-known and popularised in medicinal research, its bioactive effects are relatively lesser known. Research on cellular regulation by vitamin E and its involvement in metabolic pathways is new and emerging. Current studies on vitamin E agree that its antioxidant effect may not be enough to justify all of its effects on the vascular system. Nevertheless, vitamin E is also able to regulate gene expression and take part in signal transduction, enzymatic activities, and membrane phenomena of lipid fluxes. Several of these molecular mechanisms, as discussed below, suggest that the protective function of vitamin E against CVD is not merely due to its free radical scavenging property [40]. 

Vitamin E protects plasma membranes from free radical damage by acting as an antioxidant. However, it also protects them and promotes their repair via modulation of the membrane by a stabilization process. For instance, the chromanol head of αT binds with phospholipids to reduce mobility and fluidity in the interior layer [41]. Vitamin E is also able to stabilize the membrane of red blood cells and prevent hemolysis induced by vitamin A [23]. Furthermore, it indirectly protects membrane stability by suppressing ROS formation caused by oxidized LDL, which will be discussed in the following sections. 

Vitamin E can directly influence phospholipid metabolism by activating phospholipase A_2_ (PLA_2_), which is involved in signal transduction, and regulate the levels of lysophosphatidylcholine species (lysoPC) [42]. LysoPC are pro-inflammatory and induce platelet activation and endothelial dysfunction in the vascular system. They form complexes with vitamin E, and thus stabilize the endothelial cells. In addition, patients with obesity and type 2 diabetes fall in the CVD risk group, where high LysoPC levels in plasma were found in these individuals. It was also shown that vitamin E forms complexes with LysoPC and prevents PLA_2_ hydrolysis in rat liver lysosomal membranes [43,44]. 

In vitro studies have shown that vitamin E is able to prevent apoptosis caused by the potential cytotoxic effect of docosahexaenoic acid (DHA) in cultured cells by modulating the gene expression of the pregnane X receptor (PXR) [45] and similar heterodimeric nuclear receptors. The positive synergy between DHA and vitamin E can also affect the regulation of DP-glucuronosyltransferase 1A1 (UGT1A1) mRNA in cell detoxification and increase stearoyl-CoA desaturase (SCD) levels [46], which play a role in lipid biosynthesis. Moreover, αT decreases the production of caspase-3 in the caspase cascade, which guides the downregulation of oxidized LDL and ROS-activated apoptotic pathways in endothelial cells [47]. 

A study showed that vitamin E induces the expression of endogenous antioxidant enzymes that prevents lipid peroxidation (and hence foam cell formation). These enzymes, namely Cu/Zn superoxide dismutase (SOD) and catalase (SOC), activate PPARγ and NF-κB redox-sensitive gene regulators [48] in vascular cells. Additionally, vitamin E also decreases the release of inflammatory cytokines IL-1β, IL-8, and IL-6 and thus reduces inflammation [49] developed during excessive macrophage recruitment. Moreover, vitamin E supplementation has been shown to downregulate the expression of CD36 scavenger receptors that are responsible for the uptake of cholesterol, and consequently prevent foam cell formation [50]. Vitamin E-deficient cells also had high levels of CD36. Vitamin E upregulates the expression of PPARγ, LXRα, and ABCA1 pathways, and forms a transduction pathway for the transport and efflux of cholesterol. This results in cholesterol efflux from macrophages and the subsequent prevention of foam cell formation. Furthermore, all genes are induced in the presence of concentrated oxidized LDL, showing that vitamin E interacts with LDL via the modification of gene expression in addition to the antioxidant pathway. It can also be inferred that vitamin E supplementation can prevent the onset of hypercholesterolemia, which contributes to the onset of early atherosclerosis [51]. 

The proliferation of vascular (specifically aortic) smooth muscle cells (VSMC) is associated with the risk of vascular diseases, namely atherosclerotic lesion formation, hypercholesterolemia, and hypertension [52]. Studies have shown that αT can block VSMC proliferation via the inhibition of the isoenzyme protein kinase C (PKC) family. PKC upregulates the pathway that converts quiescent VSMC to their proliferative state. In general, PKC activates an important cell transduction pathway responsible for cell growth, proliferation, differentiation, and secretion [53]. In addition, PKC is one of the main receptors for tumour-promoting phorbol esters. αT is a specific PKC inhibitor and prevents VSMC proliferation in several ways. Experiments have shown that αT can increase phorbol ester binding to PKC. However, the overall effect of vitamin E is preventative, and phorbol-binding does not increase PKC production in cells. It is postulated that αT inhibits phosphorylation of the 80-kDa protein, which is a PKC activation marker and enzyme substrate for the signal transduction pathway. αT also prevents the translocation of phorbol-bound PKC to the membrane after its activation, and thus reduces its cellular distribution [52]. At the molecular level, vitamin E analogues prevent the activation of PKC by binding differentially to its diacylglycerol (DAG) binding sites [7]. In diabetic patients (which present a high risk of developing CVD), PKC is activated by increased DAG production. However, αT is able to inhibit PKC release by stimulating the production of DAG kinase, which phosphorylates DAG [54], and consequently lowers its levels. 

Macrophages also produce tumour necrosis factor-α (TNF-α), which leads to ROS production and the inhibition of connective tissue growth factor (CTGF). CTGF is necessary in VSMC for wound repair, tissue growth, and plaque stabilization during atherosclerotic lesion formation. αT is antagonistic to TNF-α [55] and helps in regressing CTGF downregulation induced by it [56], and subsequently prevents ROS production, inflammation, and atherosclerotic plaque build-up. Studies have also shown that oxidized LDL causes inactivation of the endothelial derived relaxation factor (EDRF) in VSMC, which is a major risk factor for CVD; this indicates EDRF release to be indirectly regulated by αT. Oxidized LDL also degrades endothelial nitric oxide (NO), which is important for vascular health and the prevention of platelet aggregation [57]. It stimulates the release of PKC through phorbol 12-myristate 13-acetate (PMA). PMA activates the G-protein, and the subsequent PKC release prevents endothelium-dependent arterial relaxation and receptor-mediated stimulation of NO production by the endothelium. The vascular incorporation of αT blocks PKC activation, and thus protects the endothelium from degeneration caused by oxidized LDL. This also provides further proof of vitamin E’s function against injury from oxidized LDL in endothelium tissue, as opposed to its antioxidant activity [30]. Further investigation showed that vitamin E prevented LDL oxidation via the release of superoxide anions (O_2_^−^) by monocytes. The enzyme NADPH oxidase is responsible for O_2_^−^ production by phagocytes. The phosphorylation of cytosolic proteins p47^phox^ by PKC and PMA is responsible for NADPH oxidase activation. However, αT blocked the translocation and phosphorylation of p47^phox^ by PMA and PKC, inhibited O_2_^−^ production by the monocytes [50,58], and consequently prevented the oxidation of LDL. 

Furthermore, oxidized LDL also activates enzyme protein kinase B (PKB/Akt) in VSMC. PKB/Akt is a major enzyme responsible for controlling various cellular processes such as cell migration, cell apoptosis, gene expression, lipid metabolism, free radical production, etc. [7]. During atherosclerotic plaque formation, increased PKB/Akt levels have been observed. Vitamin E inhibits the phosphorylation of Ser473, the key regulatory site of PKB/Akt, and subsequently regulates the production of the PKB enzyme (independent of PKC regulation). Moreover, vitamin E decreases oxidized LDL uptake by THP-1 macrophages by preventing oxidized LDL-induced Ser473 phosphorylation. This further downregulates CD36 scavenger receptors via PPAR in the ox-LDL/CD36/PKB/PPARγ pathway. As a result, it decreases overall lipid uptake and biosynthesis in cells [59]. Cholesterol biosynthesis and lipid exchange is also prevented by the binding of αT to tocopherol-associated proteins (TAP 1/2/3), which inhibit enzymes squalene epoxidase and HMG-CoA reductase [60].

Vitamin E has been established as an antioxidant that protects monounsaturated fatty acids and polyunsaturated fatty acids (MUFA/PUFA). However, it can also indirectly regulate the gene expression and production of lipid mediators regulated by MUFA/PUFA, which control membrane receptors, membrane lipid composition, signal transduction enzymes, transcription factors (including PPARγ, hepatocyte nuclear factor HNF-4α, NRF2, LXRα/β, etc.), and lipid metabolism enzymes [61]. These play a significant role in regulating the metabolism of cholesterol, carbohydrates, and triglycerides in cells [7] and ultimately determine the risk of atherosclerosis, hyperlipidemia, and hypercholesterolemia. 

**Figure 4:**
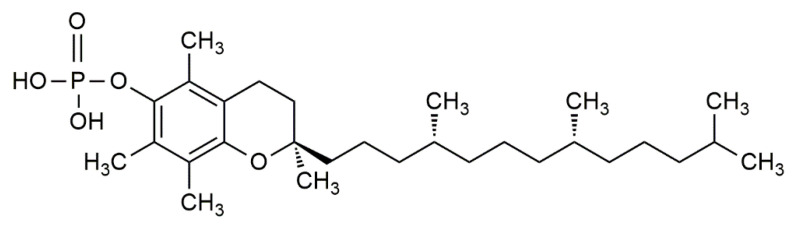
The chemical structure of α-tocopherol phosphate (αTP).

Newer studies have further laid an emphasis on the molecular effect of α-tocopherol phosphate (αTP) (Figure 4) in modulating gene expression. αTP is a natural analogue of αT that is found extensively in foods and in the human body. While αTP has no known antioxidant effect, it is shown to act as a lipid mediator in membranes, as a cofactor for enzymes, and as a receptor for various transcription factors for mRNA [62]. αTP is shown to be more potent than αT in reducing the expression of CD36 scavenger receptors and the proliferation of human THP-1 monocytes, thus lowering inflammation and atherosclerotic lesion formation. Additionally, αTP stimulates human TAP-1, which consequently interacts with the phosphatidylinositol-3-kinase (PI3K/Akt) signal transduction pathway to modulate the expression of several vascular endothelial growth factor (VEGF) genes [60,63,64]. It is shown that stimulation of VEGF genes by αTP is essential for cell and wound repair, tissue remodelling, and stimulates vascular permeability, vasculogenesis, and angiogenesis, which can prevent hypoxia in atherosclerotic regions at an early stage [63,65]. Moreover, VEGF expression can be further promoted by the production of αTP in VSMC [60]. 

CVD includes a range of disorders and conditions, with the most serious ones being CAD and stroke. Venous thromboembolism (VTE) is the third most common CVD after these two and refers to the formation of blood clotting in the deep veins of the limbic and groin region; when the clot breaks, it is circulated in the blood stream and transported to the lungs, leading to a primary lung embolism. In extreme cases, it can also cause right heart failure [66]. A clinical trial conducted on women showed that a supplemental dose of vitamin E can reduce the hazard of VTE significantly by 21%, and the hazard of unprovoked VTE by 27%. Furthermore, the risk of a primary embolism was reduced by 28%. The hypothetical reason behind this is the anticoagulation effect of vitamin E (when vitamin K levels in the body are low) as well as the inhibition of platelet adherence. Hence, it can be potentially used to prevent first or recurrent VTEs in the body [67]. A deeper look into the antithrombotic effects of vitamin E suggests that αT can prevent thrombus formation via the inhibition of platelet adherence to mononuclear cells (MNC). Oral supplementation of vitamin E given to healthy subjects suppressed PMA-mediated P-selectin expression, interactions between platelets and MNCs, platelet PKC activity, and overall agonist-induced platelet aggregation ex vivo. Since P-selectin is prothrombotic, and PKC promotes platelet-MNC interaction, vitamin E can have potential uses in the treatment of thrombosis, which reduces the risk of atherosclerosis and CAD. This also highlights the differing effects of vitamin E in arterial and venous events [68]. Further evidence showed that supplemental vitamin E downregulates gene expression for the intercellular cell adhesion molecule (ICAM-1) and vascular cell adhesion molecule-1 (VCAM-1), which are induced in the presence of elevated oxidized LDL [69], and subsequently reduces the adhesive capabilities of cellular blood components to endothelium lining of the blood vessels. In vitro studies on human cell lines also showed that αT downregulates the expression of adhesion molecules CD11b and very late antigen-4 (VLA-4), which in turn suppress the migration and adhesion of activated monocytes [70] to the endothelium. Altogether, the downregulation of ICAM-1, VCAM-1, CD11b, and VLA-4 by αT directly reduced the risk of inflammation and thrombosis in the vascular system.

Previously, it has been noted that vitamin E upregulates PLA_2_ expression. Furthermore, vitamin E can increase the expression of cyclooxygenase-1. Together, these two enzymes participate in the arachidonic acid cascade and increase prostacyclin release [42]. Prostacyclin inhibits platelet aggregation, prevents thrombosis, and acts as a potent vasodilator [5], thus reducing the risk of hypertension. Hypercholesterolemic animals showed that αT downregulates platelet aggregation by inhibiting platelet factor 4 and thromboxane B2 (TxB2) [20,71]. Blood clot formation also plays a role in increasing the rate of atherosclerosis. The hormone thrombin, released during the clotting process, is shown to have multiple effects on monocyte recruitment, endothelial cell lining, and smooth muscles, in addition to platelet aggregation. Moreover, oxidized LDL acquires procoagulant properties and further increases the rate of thrombin production [72]. Patients suffering from hypercholesterolemia and hyperlipidemia have shown high thrombin levels, as its secretion is a natural response to inflammation [73]. Additionally, it is known that platelet aggregation increases the rate of lesion formation. Since thrombin generation is further promoted by lipoproteins, vitamin E’s regulatory effect on LDL oxidization is deduced to prevent LDL-initiated thrombin production by reducing its plasma generation, hereby suggesting another mechanism by which αT prevents atherosclerosis [74] and CVD risk. 

**Figure 5:**
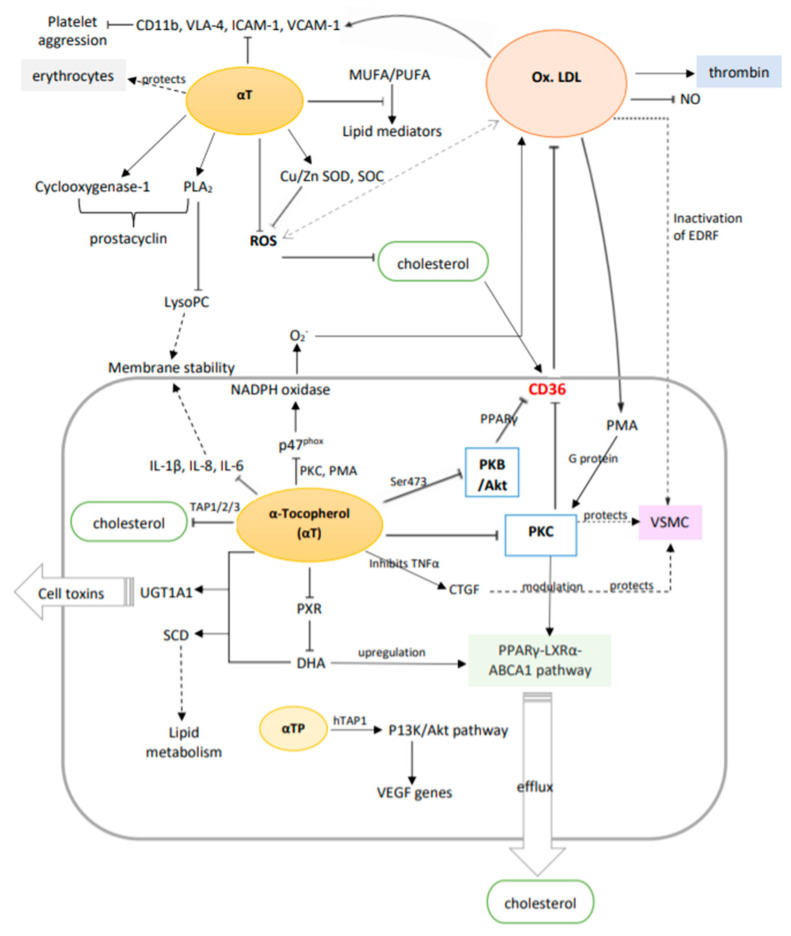
A summary of the potential molecular mechanisms of vitamin E (α-tocopherol) in vascular disease prevention via the suppression and upregulation of genes and metabolic pathways. Solid arrows: primary effect; dotted arrows: secondary effect (intermediate steps are not shown); double arrows: interactions.

The non-oxidative molecular mechanisms of vitamin E discussed above are summarised in Figure 5 and Table 3 below. 

## 4. Toxic Effects of Vitamin E

Vitamin E is shown to have many therapeutic effects, both as an antioxidant and in other cellular processes. This also raise concerns regarding the potential adverse effects of vitamin E, especially when taken in excess or in large supplemental doses. The wide availability of vitamin E supplements due to its nutritional popularity further poses the question of the plausible toxic effects of vitamin E, since high doses can induce drug-metabolism of vitamin E [75].

A clinical trial on the effect of combining selenium and vitamin E to prevent prostate cancer indicated that vitamin E can significantly increase the risk of prostate cancer incidence in men [76]. Another trial showed that while vitamin E supplementation did not affect all-cause mortality overall, significant results were seen in dose–response analyses, and a supplemental dose of >150 IU/day or a high dose (>400 IU/day) caused a progressive increase in all-cause mortality. The dose of >150 IU/day is much lower than the recommended upper limit (UL) of 1100 IU/day of synthetic and 1500 IU/day of natural vitamin E (both approximately 1000 mg/day vitamin E), and thus can be a cause of concern [77]. This evidence is supported by a randomized control trial which discouraged the supplementation of vitamin E to postmenopausal women suffering from CVD that showed high vitamin E doses increased cardiovascular mortality over a period of 2 years [78].

Vitamin E also showed significant anticlotting properties, especially in its quinone form. As mentioned previously, vitamin E has the potential to be an effective anticoagulant and inhibit clotting mechanisms that are regulated by vitamin K. This can have an opposing effect to the use of vitamin E as a treatment for CVD [79] and may be a risk factor for those on vitamin K supplementation or suffering from vitamin K deficiencies. Moreover, it has been shown that αT inhibits the enzyme glutathione S-transferase (GST) in human due to its preferential binding over GST with proteins like haemoglobin and albumin. All isozymes of GST found in the cytosol of hepatic cells were also inhibited by αT. GST inhibition can lead to the loss of its detoxification ability of electrophilic compounds, which can cause damage to proteins, lipids, and DNA, and further progress cancer development, neoplastic diseases, and neurodegenerative diseases [80]. In fact, early studies on vitamin E and cancer prevention argued that vitamin E does not show protective effects against the onset of lung, prostate, and colorectal cancer, and might even increase the risk of haemorrhagic stroke in men [81]. In reactions with low radical flux or the presence of transition metals, high doses of vitamin E can play the role of a pro-oxidant. It results in the production of α-tocopherol radicals which further facilitate the peroxidation of lipids in blood vessels. While its pro-oxidation can be inhibited by other antioxidants such as vitamin C, the presence of a mixture of high vitamin E and vitamin C can be potentially hazardous due to the pro-oxidant-rich environment created in atherosclerotic plaques [82].

Nonetheless, the potential cytotoxic effects of supplemental doses of vitamin E have raised concern among researchers and suggested the need to define a lower UL for vitamin E. There is also a need for more investigation into vitamin E’s interaction with other micronutrients in order to study their combined roles in human physiology [75].

## 5. Concluding Remarks

This review viewed vitamin E as an important micronutrient for vascular health. It is found in common food sources, namely routinely consumed edible oils, nuts, and seeds, which makes it rare for one to suffer from vitamin E deficiency. Over several decades, vitamin E has gained popularity as a micronutrient as it acts as a major deterrent of atherosclerosis, which is the biggest cause of CVD in humans.

The earliest research on vitamin E established its roles as a fat-soluble antioxidant due to lowered rates of atherosclerosis and an overall reduction in cardiovascular mortality observed in randomized-controlled trials. While these studies could not pinpoint the exact reasons behind vitamin E’s effect, it has been well-established in the past to be an effective radical scavenger to prevent LDL oxidation and foam cell formation, and subsequently prevents the formation of atherosclerotic lesions, inhibits plaque build-up and stenosis, and lowers hypertension—which are all major risk factors of poor vascular health. While some studies also claimed that vitamin E did not show any improvement to vascular health, the relationship seemed causal, and most studies could not hypothesize valid reasons behind the potential anti-protective effects of vitamin E.

Studies also underlined various cellular mechanisms that allows αT to prevent the oxidation of LDL, in addition to its antioxidant property. Vitamin E inhibits other factors that promote vascular degeneration and atherosclerotic lesion formation. It not just protects arteries from atherosclerosis and platelet aggregation, but also helps in the overall prevention of venous thromboembolism, vascular cell toxicity, endothelial membrane apoptosis, hypercholesterolemia, angiogenesis, thrombosis, and hypertension. Moreover, it regulates cholesterol efflux, increases lipid metabolism, regulates vascular smooth muscle contraction, and increases wound healing and cell repair and growth in vascular tissues. These findings prove that the vitamin has regulatory effects beyond the prevention of LDL oxidation. This claim is further purported by the fact that vitamin E deficiency leads to several disorders that occur not just from an ‘increase in oxidative stress’ in humans [50]. Accordingly, the regulatory role of vitamin E at the molecular level should be considered when relating its association with human vascular health.

A shortcoming of present research that is highlighted in this review is the lack of animal and human studies on the molecular mechanisms by which αT protects vascular function. While in vitro studies contend that αT has a host of regulatory effects on the vascular endothelium, these claims need to be validated by in vivo studies as well. Moreover, there are many conflicting studies about the exact function of vitamin E. This is mainly because of the difficulties faced in identifying the exact form of vitamin E (such as the type of tocopherol vitamer) that participates in gene expression and regulation [50]. Deeper research will also allow more coherence in the conclusions drawn by several present and ongoing studies on vitamin E’s effect on the vascular system [75].

There are no direct harmful effects of taking supplemental doses of tocopherol, while small, regular supplementation can potentially be beneficial for people suffering from vascular diseases. In fact, research shows promising effects of the potential therapeutic use of vitamin E for the prevention and treatment of CVD. Nonetheless, caution must be taken to prevent overdosing and to ensure that vitamin E works in harmony with other important vitamins such as C, A, and K, and does not cause any potential cytotoxic effects.

## Figures and Tables

**Table 3:** A summary of the molecular mechanisms of vitamin E in vascular diseases.

Pathological/Biological Condition	Effect	Target Tissues	Reference
Hemolysis	↑ membrane stability, ↓ phospholipid fluidity	Blood	[41]
Membranal instability	Activates PLA_2_, regulates and forms complexes with LysoPC	Vascular endothelium	[43]
Membrane apoptosis	Regulates PXR and other heterodimeric nuclear receptors’ expression	Vascular endothelium	[46]
Cell toxicity	Interacts with DHA to regulate UGT1A1 mRNA expression	Vascular endothelium	[46]
Hypercholesterolemia	Interacts with DHA to regulate SCD levels that improve lipid metabolism	Arteries	[46]
Cell apoptosis	↓ caspace-3 production	Vascular endothelium	[47]
Atherosclerosis	↑ Cu/Zn SOD, SOC production	Vascular endothelium	[48]
Inflammation	↓ cytokines IL-1β, IL-8, IL-6	Vascular endothelium	[49]
Hypercholesterolemia, atherosclerosis	↓ CD36 expression, ↑ PPARγ-LXRα-ABCA1 pathway (in the presence of ox-LDL) which ↓ cholesterol and × foam cells	Arteries	[51]
Atherosclerosis, hypercholesterolemia, hypertension	× PKC, ↓ VSMC proliferation, protects endothelial NO release and vascular relaxation, × ox-LDL and PMA release	Vascular endothelium, vascular muscles	[30,52,53]
CVD caused from diabetes	↓ DAG by ↑ DAG kinase which × PKC	Vascular endothelium	[54]
Atherosclerosis, inflammation	× TNF-α which ↑ CTGF in VSMC	Vascular muscles	[56]
Atherosclerosis	× phosphorylation of p47^phox^ by PMA and PKC results in × NADPH oxidase which ↓ O_2_^−^ production and hence ↓ ox-LDL	Vascular endothelium	[58]
Atherosclerosis	↓ PKB/Akt production, which ↓ CD36 via the ox-LDL/CD36/PKB/PPARγ pathway	Vascular endothelium, vascular muscles	[7,59]
Hypercholesterolemia, atherosclerosis, hyperlipidemia	× MUFA or PUFA peroxidation, regulates various lipid mediators	Arteries, vascular endothelium	[7,61]
Atherosclerotic lesions, arterial inflammation	αTP ↓ CD36 expression, THP-1 monocyte proliferation	Arteries	[63]
Hypercholesterolemia	↓ cholesterol synthesis by binding to TAP1/2/3	Arteries	[64]
Atherosclerosis	αTP modulates VEGF genes expression through the PI3K/Akt pathway which ↑ cell repair, wound healing, vascular permeability, vasculogenesis, angiogenesis, and × hypoxia	Arteries	[60,63,65]
VTE	↓ hazard, anticoagulation and ↓ platelet clotting	Blood, lungs	[67]
Thrombosis	× platelet aggregation by × platelet-MNC interaction, PKC activity, PMA-mediated P-selectin expression	Blood	[68]
Inflammation, thrombosis	↓ ICAM-1 and VCAM-1, which ↓ blood cell adhesion to vessels, ↓ CD11b, VLA-4	Arteries, veins	[5,70]
Thrombosis, hypertension	↑ PLA_2_ and cyclooxygenase-1, which ↑ prostacyclin, which in turn ↑ vasodilation and ↓ platelet aggregation	Arteries, veins	[5]
Atherosclerosis, hyperlipidemia	↓ platelet aggregation by ↓ LDL-initiated thrombin hormone production	Arteries	[74]

↑: increases/upregulates; ↓: reduces/downregulates; ×: suppresses; PLA2: phospholipase A2; LysoPC: lysophosphatidylcholine species; PXR: pregnane X receptor; DHA: docosahexaenoic acid; UGT1A1: DP-glucuronosyltransferase 1A1; SCD: stearoyl-CoA desaturase; SOD, SOC: superoxide dismutase, caspace; αTP: α-tocopherol phosphate; CD36: CD36 scavenger receptor; PKC: enzyme protein kinase C; VSMC: vascular smooth muscle cell; PKB/Akt: protein kinase B; O_2_^−^: superoxide anion; NO: nitric oxide; PMA: phorbol 12-myristate 13-acetate; DAG: diacylglycerol; TNF-α: tumor necrosis factor-α; CTGF: connective tissue growth factor; ox-LDL: oxidized low-density lipoprotein; MUFA/PUFA: unsaturated fatty acids; TAP1/2/3: tocopherol-associated proteins; VEGF: vascular endothelial growth factor; PI3K/Akt: phosphatidylinositol-3-kinase; VTE: venous thromboembolism; MNC: mononuclear cells; ICAM-1: intercellular cell adhesion molecule; VCAM-1: vascular cell adhesion molecule-1.
